# Functionalized Gold Nanoparticles Suppress the Proliferation of Human Lung Alveolar Adenocarcinoma Cells by Deubiquitinating Enzymes Inhibition

**DOI:** 10.1021/acsomega.3c05452

**Published:** 2023-10-20

**Authors:** Bashiru Ibrahim, Taiwo Hassan Akere, Swaroop Chakraborty, Eugenia Valsami-Jones, Hanene Ali-Boucetta

**Affiliations:** † Nanomedicine, Drug Delivery & Nanotoxicology (NDDN) Lab, School of Pharmacy, College of Medical and Dental Sciences, University of Birmingham, Birmingham B15 2TT, U.K.; ‡ School of Geography, Earth and Environmental Sciences, College of Life and Environmental Sciences, University of Birmingham, Birmingham B15 2TT, U.K.

## Abstract

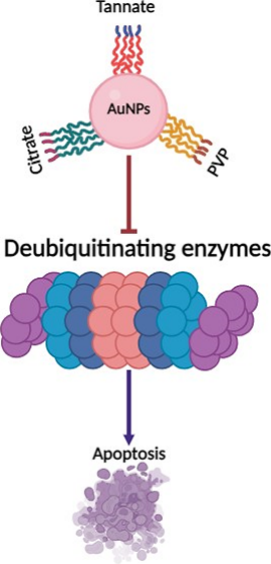

Functionalized gold nanoparticles (AuNPs) are widely used in therapeutic applications, but little is known regarding the impact of their surface functionalization in the process of toxicity against cancer cells. This study investigates the anticancer effects of 5 nm spherical AuNPs functionalized with tannate, citrate, and PVP on deubiquitinating enzymes (DUBs) in human lung alveolar adenocarcinoma (A549) cells. Our findings show that functionalized AuNPs reduce the cell viability in a concentration- and time-dependent manner as measured by modified lactate dehydrogenase (mLDH) and 3-[4,5-dimethylthiazole-2-yl]-2,5-diphenyltetrazolium bromide (MTT) assays. An increased generation of intracellular reactive oxygen species (ROS) and depletion of glutathione (GSH/GSSG) ratio was observed with the highest AuNP concentration of 10 μg/mL. The expression of DUBs such as ubiquitin specific proteases (USP7, USP8, and USP10) was slightly inhibited when treated with concentrations above 2.5 μg/mL. Moreover, functionalized AuNPs showed an inhibitory effect on protein kinase B/mammalian target of rapamycin (PI3K/AKT/mTOR) and wingless-related integration site (Wnt) signaling proteins, and this could further trigger mitochondrial related-apoptosis by the upregulation of caspase-3, caspase-9, and PARP in A549 cells. Furthermore, our study shows a mechanistic understanding of how functionalized AuNPs inhibit the DUBs, consequently suppressing cell proliferation, and can be modulated as an approach toward anticancer therapy. The study also warrants the need for future work to investigate the effect of functionalized AuNPs on DUB on other cancer cell lines both in vitro and in vivo.

## 1 Introduction

Engineered nanoparticles (ENPs), such as AuNPs, have shown promising applications in the field of nanoscience due to their small size (typically between 1 and 100 nm) and large surface area to volume ratio, which gives nanoparticles' distinguishable properties compared to their bulk materials.^1−3^ AuNPs are commonly functionalized with different capping agents such as citrate, polyethylene glycol (PEG), polyvinylpyrrolidone (PVP), tannic acid, carboxylic acid, carbonated, and polystyrene to prevent aggregation, improve biocompatibility, and stability.^4,5^ Functionalized AuNPs are widely used in cancer research, photothermal therapy,^6^ bioimaging, and drug delivery due to their low cytotoxicity.^7,8^ Oxidative stress imbalance, membrane damage, genotoxicity, apoptosis, necrosis, and activation of signaling pathways such as protein kinase B (AKT), wingless-related integration site (WNT), mitogen-activated protein (MAPK), and c-Jun N-terminal kinase (JNK) were described as possible mechanisms of toxicity associated with the exposure to functionalized AuNPs.^9−11^ In addition, the influence of functionalization on the toxicity of AuNPs has been demonstrated in several studies.^12,13^ For instance, a previous study^14^ found that AuNPs functionalized with polyethylene glycol (PEG) suppressed the proliferation of cells through ROS generation and reduction in mitochondrial activity.^14^ In A549 cells, AuNPs functionalized with citrate showed higher toxicity in comparison to polyethylene imine functionalized AuNPs.^15^ In another study, the role of functionalization on the genotoxicity and cytotoxicity in BEAS-2B cells was investigated using carboxylate, ammonium, and PEG. The study revealed that AuNPs functionalized with ammonium were more genotoxic and cytotoxic compared to AuNPs functionalized with carboxylate and PEG.^16^ However, there are no reports on the effects of different functionalized AuNPs on deubiquitinating enzymes (DUBs) and their utility in understanding anticancer pathways. DUBs are an important group of enzymes that play a significant role in cancer pathogenesis, development, and proliferation by removing attached ubiquitin from the substrate.^17,18^ They are classified into ubiquitin c-terminal hydrolases (UCHs), ubiquitin specific proteases (USPs), ovarian tumor proteases (OTUs), machado Joephin domain proteases (MJDs), monocyte chemotactic protein induced proteins (MCPIPs), and JAB1/MPN/Mov34 metalloenzyme (JAMM).^19−22^ Among these DUBs, USPs, and UCHs are the most well-studied as they are highly overexpressed in human cancers, suggestive of their roles in tumor progression.^23,24^ It has been found that cell survival in liver, breast, and colorectal cancers was promoted by an elevated level of USPs through simultaneously inhibiting apoptotic proteins and promoting tumor metastasis.^25,26^ UCHL-1 overexpression has also been positively correlated with development, invasiveness, and chemotherapy resistance in some cancers including pancreatic, myeloma, neuroblastoma, nonsmall cell lung cancer (NSCLC), prostate, and lymphoma.^27,28^ The overarching aim of this study is to understand the effects of tannate, citrate, and PVP functionalized 5 nm AuNPs on DUBs with a particular focus on USPs and UCHL-1 in A549 cells proliferation and toxicity. The study further hypothesized if the inhibition of these DUBs could lead to the downregulation of PI3K/AKT/mTOR, Wnt signaling pathway related proteins, and activation of mitochondrial apoptosis. The expression levels of DUBs, PI3K/AKT/mTOR, and Wnt signaling-related proteins in noncancerous (MRC-5) and cancerous cells (A549) without AuNPs were also evaluated. Five nanometers functionalized AuNPs were selected for this study because the recent literature is currently biased toward larger functionalized AuNPs which are easier to functionalize and characterize but also due to a lack of reliable data on the toxicity of 5 nm AuNPs.

## 2 Materials and Methods

### 2.1 Materials and Reagents

5 nm AuNPs functionalized with tannate, citrate, and PVP, all purchased from NanoComposix (San Diego, CA, USA), were used for this study. Phosphate-buffered saline (PBS), F-12 nutrimix media, penicillin/streptomycin, 0.25% trypsin-EDTA, fetal bovine serum (FBS), and RPMI 1640 were purchased from Thermo Fisher Scientific (Paisley, UK). CytoTox 96 nonradioactive cytotoxicity and GSH/GSSG-Glo luminescence assay kits were acquired from Promega (Southampton, UK). Menadione, staurosporine, dimethyl sulfoxide (DMSO), tris-base, glycine, MTT (3-(4,5-dimethylthiazol-2-yl)-2,5-diphenyltetrazolium bromide), dichlorofluorescin diacetate (DCFDA), and sodium chloride were purchased from Sigma-Aldrich (Dorset, UK). Bovine serum albumin (BSA), acrylamide/bis-acrylamide, and ponceau were bought from Alfa Aesar (Lancashire, UK). The Alexa fluor 488 Annexin V/PI cell death reagent kit was purchased from Thermo Fisher Scientific (Paisley, UK). The PVDF membrane and ECL were purchased from GE HealthCare (Buckinghamshire, UK). USP7, USP8, USP10, UCHL-1, AKT, p-AKT, mTOR, GSK-3β, β-catenin, p-β-catenin, GAPDH, caspase-3, caspase-9, and PARP primary antibodies were purchased from Cell Signaling Technology (CST) (London, UK).

### 2.2 Physicochemical Characterization of Various Functionalized AuNPs

The hydrodynamic diameter, polydispersity index (PDI), and zeta potential of 5 nm functionalized AuNPs (Tannate, citrate, and PVP) at a concentration of 10 μg/mL were measured in ultrapure water (UPW), phosphate buffer saline (PBS), free culture media (FCM), and cell culture media (CCM) supplemented with 10% FBS and 1% penicillin/streptomycin using dynamic light scattering (DLS) (Malvern Instruments Ltd., Worcestershire, UK) at 25 °C. A total of 1 mL of the dispersed AuNPs was pipetted into a disposable polystyrene cuvette (Bohemia, NC, USA) for size and PDI measurement. For zeta potential, the dispersions were pipetted into a capillary cell cuvette (Malvern Instruments Ltd., Worcestershire, UK). The morphology and size distribution of the nanoparticles were studied using Transmission Electron Microscopy (TEM) (JEOL Ltd., UK) instrument operating at an accelerating voltage of 80 keV (JEOL 1400 TEM, University of Birmingham, Centre for Electron Microscopy). A freshly prepared 10 μL portion of AuNPs (10 μg/mL) dispersed in UPW was deposited onto a copper 300 Mesh TEM grid (EM Resolutions Ltd., Sheffield, UK) and allowed to dry for 2 h in a safety cabinet before observation by TEM.

### 2.3 Cell Culture

Lung epithelial cancer cell lines (A549) were purchased from the American type culture collection (ATCC) and cultured in F-12 nutrimix media supplemented with 10% FBS, 1%100 μg/mL antibiotics (penicillin-streptomycin) in a humidified incubator with 5% CO_2_ at 37 °C. The cells were passaged when the confluence reached 70–80%.

### 2.4 Cell Viability Assays

Percentage cell viability of the A549 cells were assessed using MTT and mLDH assays by Promega CytoTox 96 non-radioactive cytotoxicity assay as reported.^29^ Briefly, for the mLDH assay, A549 cells were seeded on 96-well plates at a density of 7000 cells/well and allowed to adhere overnight. The cells were then exposed to 5 nm tannate, citrate, and PVP functionalized AuNPs at concentrations ranging from 1.25–35 μg/mL, and untreated cells (negative control) were incubated with CCM while the positive control was incubated with 20% DMSO for 24 and 48 h. After the incubation period, the cells were then lysed with 0.9% Triton X100 and incubated for another 1 h at 37 °C. The lysates were collected in Eppendorf tubes and centrifuged for 5 min at 4 °C, 50 μL of the supernatants was transferred to a new 96-well plate, and 50 μL of substrate mixture was added and incubated for 15 min at room temperature covered with foil. After the incubation, 50 μL of stopped solution was then added, and the absorbance of each sample was measured using a FLUOstar Omega microplate reader (BMG LABTECH Ltd., Aylesbury, UK) at 490 nm emission wavelength. For the MTT Assay, post nanoparticle exposure, 120 μL of MTT (5 mg/mL in PBS) was added to each well and incubated for 4 h at 37 °C. The MTT solution was replaced by adding 200 μL of DMSO into each well for 10–15 min to solubilize the formazan crystals formed by cells in the dark with gentle agitation using a shaker. The absorbance was recorded using a FLUOstar Omega microplate reader (BMG LABTECH Ltd., Aylesbury, UK) at a 570 nm emission wavelength. The percentage cell viability was calculated using the following formula:
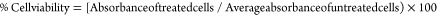


### 2.5 Cell Recovery

To evaluate A549 cells recovery capacity upon exposure to 5 nm functionalized AuNPs. A549 cells were seeded into a 96-well plate at a density of 7000 cells/well overnight to adhere and then exposed to 1.25–10 μg/mL of AuNPs for 24 h. After the exposure, the cells were washed with PBS three times, and 100 μL of fresh growth media was added every 24 h. The number of viable cells recovered was measured on days 1, 2, 3, and 4 using the mLDH according to Section 2.4.

### 2.6 Oxidative Stress Parameters

The generation of intracellular reactive oxygen species (ROS) in A549 cells after treatment with different functionalized 5 nm AuNPs for 24 and 48 h at concentrations ranging from 1.25–10 μg/mL were measured using 2,7-dichlorofluorescin diacetate (DCFH-DA).^30^ Intracellular glutathione was determined using the GSH/GSSG-Glo assay kit as instructed by the manufacturer (Promega, Southampton, UK). Briefly, A549 cells were seeded on 96-opaque well plates and allowed to attach for 24 h. The cells were then treated with different functionalized AuNPs for 24 and 48 h. After the treatment, cells were washed with 1% PBS three times to remove any remaining AuNPs. The cells were lysed with 50 μL of either total glutathione or oxidized glutathione reagent and placed on a shaker for 5 min at room temperature, and 50 μL of the luciferin generation reagent was added to all the wells and incubated for another 30 min at room temperature. Finally, 100 μL of luciferin detection reagent was added and equilibrated for 15 min at room temperature, followed by luminescence reading using a FLUOstar Omega microplate reader (BMG LABTECH Ltd., Aylesbury, UK). The ratio of glutathione content was calculated by using the relative light units (RLU) recorded by the microplate reader.

GSH/GSSG ratio were calculated as follows:
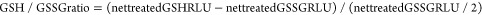


### 2.7 Mitochondrial Membrane Potential

Change in MMP induced by different functionalized AuNPs on A549 cells was evaluated according to the method described.^31^ Briefly, A549 cells were seeded on a 96-well plate at a density of 7000 cells/well and allowed to attach overnight. The media was then removed, and cells were exposed to different functionalized AuNPs at a concentration range of 1.25–10 μg/mL diluted in CCM for 24 h. After the incubation, 50 μL of mitochondrial staining solution was added to each well and the cells were incubated for 30 min under normal cell culture conditions. Afterward, the medium was removed, and cells were counterstained with 100 μL of Hoechst 33342 staining solution and incubated for another 15 min at normal room temperature. The cells were then washed with 100 μL of PBS three times, and 200 μL of PBS was added to each well. Finally, the MMP changes were measured by using two different techniques. First, the fluorescence intensity was captured using a FLUOstar Omega microplate reader (BMG LABTECH Ltd., Aylesbury, UK) in a time-resolved fluorescence manner. Second, the quality of fluorescence intensity was captured using EVOS microscopy equipped with an appropriate filter for DAPI and GFP at 20× magnification.

### 2.8 Annexin V-FITC/PI Apoptosis Assay

To confirm whether the exposure of 5 nm AuNPs induced apoptosis, an annexin V-FITC/PI staining assay was conducted according to the manufacturer’s instructions. Briefly, A549 cells were seeded at a density of 2.5 × 10^5^ cells/well in 6-well plates and treated with fresh media containing different functionalized AuNPs at a concentration range of 1.25–10 μg/mL for 24 h. One μM Staurosporine and 10% DMSO for 6 h was used as a positive control for apoptosis and necrosis, respectively. The samples were then analyzed using BD LSRFortessa X20 flow cytometry (BD Biosciences, Franklin Lakes, NJ, USA) by acquiring at least 10,000 events. Three independent experiments were carried out and the data was analyzed using FlowJo software.

### 2.9 Western Blotting

A549 cells were seeded on six well plates at a density of 2.5 × 10^5^ cells/well and allowed to adhere overnight at 37 °C and 5% CO_2_. Cells were then treated with different functionalized AuNPs (tannate, citrate, and PVP) dispersed in CCM at a concentration range of 1.25–1 0 μg/mL for 24 h. The cells were lysed with NP40 lysis buffer containing mercaptoethanol and phenylmethylsulfonyl fluoride (PMSF). Protein concentration in the samples was then measured using the Bradford assay kit according to the manufacturer’s instructions. Proteins (20–30 μL) were loaded in either 10 or 12% SDS-PAGE (Bio-Rad, Ltd., Watford, UK) and separated according to their molecular weight connected to the power supply (160 V) for 1 h. The proteins were then transferred to the PVDF membrane (GE Healthcare, UK) in a running buffer prepared from NaCl, Tris-Base, and 20% methanol connected to a power supply with an accelerating voltage of 85 V for 1:45 h. The membrane was blocked for 1 h in TBST with 5% fat milk at room temperature and incubated with primary antibodies of interest overnight at 4 °C on a shaker with gentle agitation. The membrane was then washed with TBST three times and subsequently incubated with secondary antibodies at room temperature for 1 h. The ECL reagent was added to the membrane after washing three times, and protein bands were detected using G:BOX Chem XX6/XX9 (Syngene, Cambridge UK).

### 2.10 Statistical Analysis

Statistical analyses were performed using GraphPad Prism software, version 9.0. BD LSR-Fortessa X20 flow cytometry data were analyzed by FlowJo software version 10.7.1 (Ashland, OR, USA). Data were expressed as the means ± the standard deviation (SD) for at least three independent experiments. One-way analysis of variance (ANOVA) was used to calculate the statistical significance difference between the control and AuNP-treated groups, followed by the Bonferroni post hoc test for multiple comparisons.

## 3 Results

### 3.1 Physicochemical Characterization of Different Functionalized AuNPs

**Figure 1:**
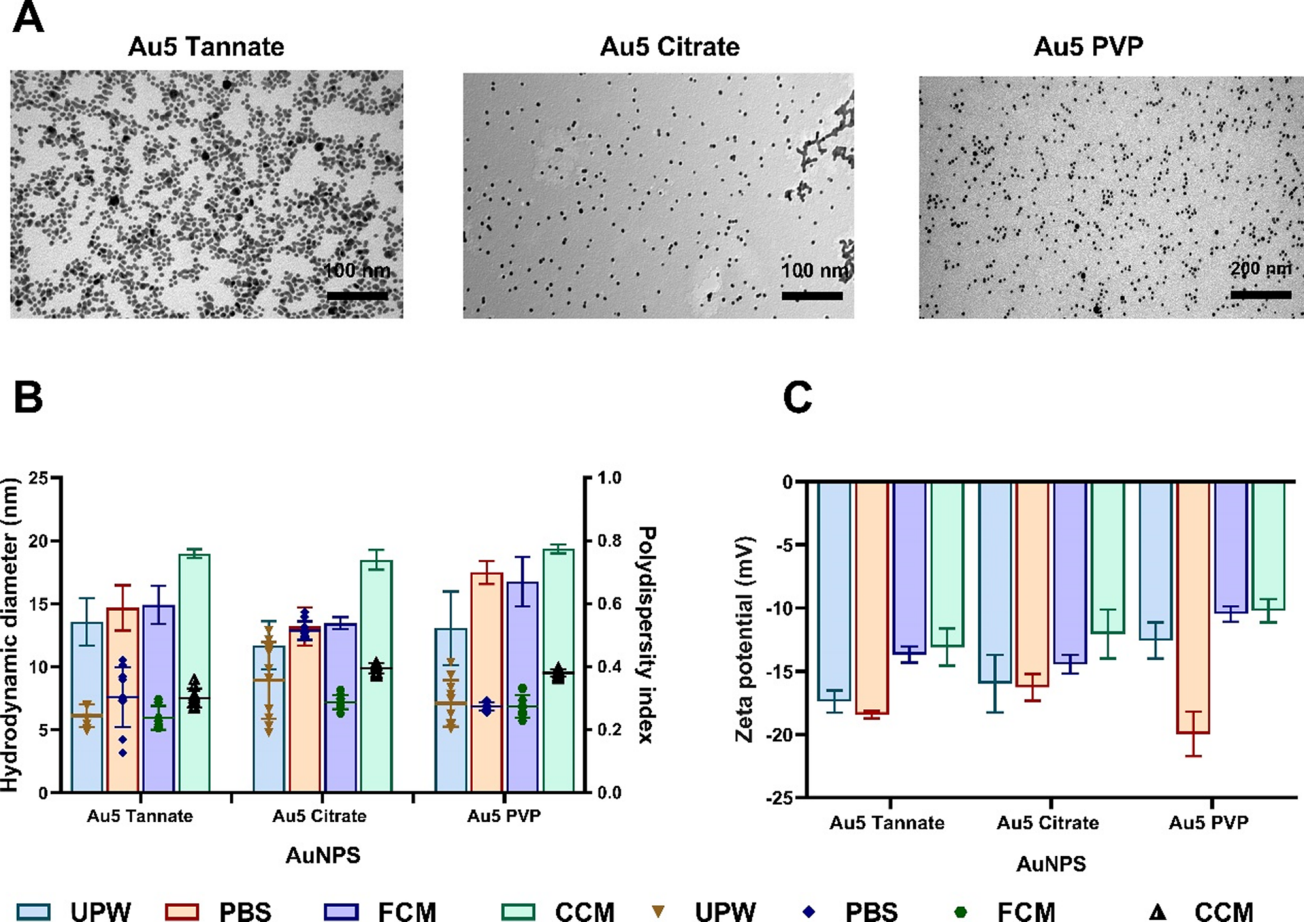
Physicochemical characterization of different surface functionalized AuNPs. (A) Transmission electron images of 5 nm AuNPs dispersed in UPW. (B) Hydrodynamic diameter and polydispersity index of 5 nm AuNPs dispersed in UPW, PBS, FCM, and CCM with 10% FBS. (C) Zeta potentials of AuNPs dispersed in UPW, PBS, FCM, and CCM. The plotted graphs represent the means ± standard deviation (SD) of ten different measurements. (Scale bar: 100 nm was used for tannate and citrate functionalized AuNPs. 200 nm was used for PVP functionalized AuNPs).

It is crucial to study the physicochemical properties of the NPs to understand the interaction of different surface functionalized AuNPs with the biological milieu. The functionalized AuNPs were first analyzed using different characterization techniques such as TEM, dynamic light scattering (DLS), and ultraviolet spectroscopy (UV–vis). Figure 1 shows the size distribution, surface morphology, aggregation, and particle dispersion of each of the surface functionalized AuNPs dispersed in UPW. TEM images of all AuNPs showed monodispersed spherical morphology with different average core size distributions closer to the size distribution stated by the manufacturer. Tannate, citrate, and PVP functionalized AuNPs showed around 6.3 ± 3.02, 5.5 ± 0.34, and 5.8 ± 1.02 nm, respectively, as calculated using ImageJ from the TEM images (Figure 1A). The hydrodynamic diameter and polydispersity index (PDI) in ultrapure water (UPW), phosphate buffer saline (PBS), free culture media (FCM), and complete culture media (CCM) supplemented with 10% fetal bovine serum were measured using DLS at a temperature of 25 °C. DLS measurements revealed that the hydrodynamic diameter of 5 nm tannate functionalized AuNPs was very close in UPW, PBS, and FCM but higher in CCM, as shown in Figure 1B. Similar observations were seen with the 5 nm citrate functionalized AuNPs in which the hydrodynamic diameter was similar in PBS and FCM, although slightly smaller in UPW and higher in CCM as observed with the tannate functionalized AuNPs (Figure 1B). Interestingly, the hydrodynamic diameter of 5 nm PVP functionalized AuNPs was larger in PBS, FCM, and CCM than UPW as highlighted, as shown in Figure 1B. Overall, all three surface functionalized AuNPs showed a slight increase in size in PBS, FCM, and CCM compared to UPW. Notably, DLS demonstrated that all the particles have a PDI of less than 0.6 in UPW, PBS, FCM, and CCM (Figure 1B).

Moreover, all AuNPs showed a negative surface charge (zeta potential) in the different media including 5 nm tannate, citrate, and PVP functionalized AuNPs, as shown in Figure 1C. In addition, the absorption spectra of the AuNPs were characterized by UV–vis in the 300–800 nm range. At this wavelength range, AuNPs showed a distinct optical feature commonly referred to as localized surface plasmon resonance (LSPR) with the position and intensity of the LSPR band depending on the size and surface morphology of nanoparticles.^32−34^ For the 5 nm citrate, PVP, or tannate functionalized AuNPs, the absorption peak was observed around 517–518 nm, which indicates the formation of small-size AuNPs (Figure S1 in the Supporting Information).

### 3.2 Cytotoxicity of A549 Cells Exposed to Different Functionalized AuNPs

**Figure 2:**
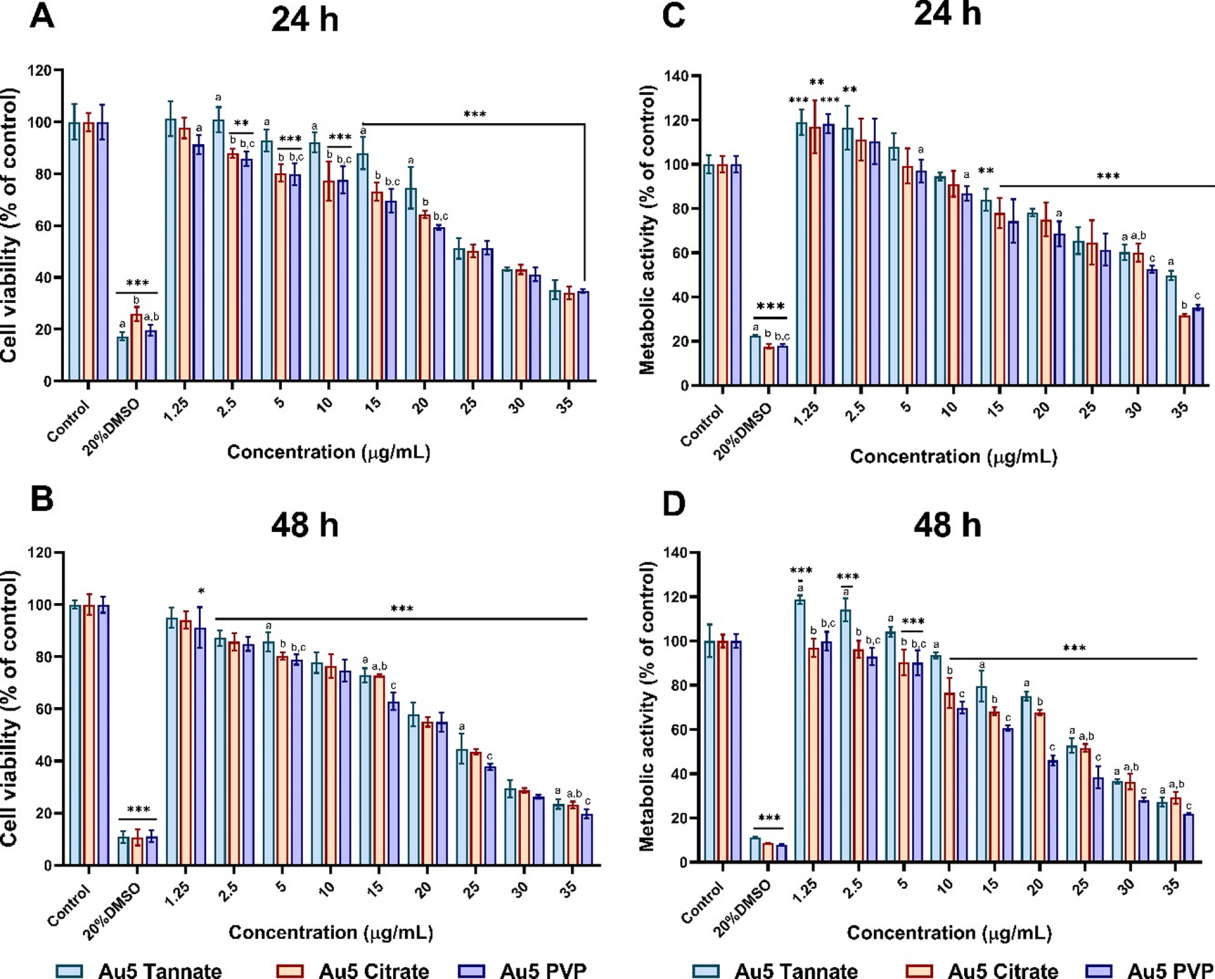
Percentage cell viability and metabolic activity of A549 cells treated with 1.25–35 μg/mL of 5 nm AuNPs coated with tannate, citrate, and PVP with 20% DMSO used as a positive control measured using mLDH and MTT assay. (A) Percentage of cell viability after 24 h. (B) Percentage of cell viability after 48 h. (C) Metabolic activity after 24 h. (D) Metabolic activity after 48 h. The plotted graphs represent the means ± standard deviation (SD) of six independent experiments. Bars with an asterisk (*) show statistical differences (*p < 0.05, **p < 0.01, ***p < 0.001) when compared with the control. Data with different letters above the bars represent the statistically significant difference (*p* < 0.05) between the functionalized AuNPs.

Cellular response of A549 cell exposed to 5 nm tannate, citrate, and PVP functionalized AuNPs at a range of concentrations (1.25–35 μg/mL) was evaluated using mLDH and MTT assay for 24 and 48 h. As seen in Figure 2A, there is a clear decrease in cell viability of A549 cells in a concentration-dependent manner with all the forms of AuNPs at 24 h. While AuNPs functionalized with tannate at concentrations of 1.25–10 μg/mL did not induce any significant reduction in A549 cell viability after 24 h, a higher reduction in cell viability (64.82%) was seen at 35 μg/mL. On the other hand, 5 nm citrate functionalized AuNPs started to show a reduction in percentage cell viability at 2.5 μg/mL of AuNPs with the highest concentration showing 63% cell viability. Likewise, treatment with 35 μg/mL of PVP functionalized AuNPs for 24 h showed a reduction in cell viability similar to that observed with the same concentration of tannate functionalized AuNPs. Further incubation for 48 h revealed similar dose-dependent effects on cell viability for all three functionalized AuNPs. In comparison to 24 h, the result showed a decrease in percentage cell viability after treatment with 1.25 μg/mL for all three AuNPs (Figure 2B). As expected, the higher concentration (35 μg/mL) of all AuNPs showed more cytotoxicity to A549 cells after 48 h incubation.

The study then focused on whether the three functionalized 5 nm AuNPs might affect metabolic activity in A549 cells. As shown in Figure 2C,D, the cytotoxicity of A549 cells was not only dependent on AuNP concentrations but also on the incubation time. Moreover, the higher concentration of all AuNPs decreased A549 metabolic activity at all time points. Overall, for the same concentration, 5 nm PVP functionalized AuNPs caused significant (*p* < 0.05) cytotoxicity compared to tannate and citrate functionalized AuNPs. To understand if the effects seen with the functionalized AuNPs are not due to the functionalization alone, 2 mM tannate, citrate, and PVP stabilizer were prepared in UPW which was equivalent to the concentration used in AuNPs. A549 cells were further exposed to these stabilizing agents (tannate, citrate, and PVP) at concentrations 0.125–1 mM diluted in CCM which is equivalent to 1.25–10 μg/mL for 24 h. At the end of the exposure, all three stabilizing agents exhibited a dose-dependent cytotoxicity to A549 cells (Figure S2 in the Supporting Information). With 0.125 mM, tannate, and citrate showed no sign of cytotoxicity compared to PVP which decreased the percentage cell viability to 84.6 ± 1.89%. When the concentrations of both citrate and PVP agents are doubled to 0.25 mM, the percentage cell viability was 87.83 ± 5.0% and 73.1 ± 0.73%, respectively, compared to tannate 100.75 ± 1.65%. With 0.5 and 1 mM, on the other hand, all the stabilizing agents significantly (*p* < 0.001) reduced A549 cell viability with PVP having the most significant (*p* < 0.05) effects (Figure S2 in theSupporting Information). This shows that there was a positive correlation between the stabilizing agents used in the NPs and toxicity. Hence, for the rest of the experiments, a concentration range of 1.25–10 μg/mL for all the functionalized AuNPs was used.

Based on the mLDH and MTT assays, the inhibitory concentrations (IC_50_) of tannate, citrate, and PVP functionalized AuNPs (5 nm) were calculated from the linear regression curve using GraphPad Prism after 24 and 48 h, as shown in Table 1. IC_50_ represents an inhibitory concentration of AuNPs that decreases the percentage of cell viability to 50% after 24 or 48 h incubation. The IC_50_ values obtained by mLDH are lower than the values obtained by the MTT assay at all incubation periods, which could indicate that the decrease in cell viability observed by AuNPs is greater than that observed with the metabolic activity. Furthermore, as surface functionalized AuNPs are compared, it can be seen that both tannate- and citrate functionalized 5 nm AuNPs demonstrated low IC_50_ values in A549 cells compared to PVP functionalized AuNPs.

**Table 1:** Calculated IC_50_ of 5 nm Functionalized AuNPs in A549 Cells^a^

5 nm AuNPs	IC_50_ concentration (μg/mL)			
	mLDH		MTT	
	24 h	48 h	24 h	48 h
Tannate	28.00 ± 0.83	23.33 ± 1.19	34.58 ± 1.45	25.84 ± 0.755
Citrate	27.23 ± 0.42	22.98 ± 0.61	30.43 ± 2.23	23.96 ± 0.436
PVP	26.27 ± 0.65	20.96 ± 1.50	29.00 + 1.78	17.92 ± 0.787

a ***Note:*** The inhibitory concentration of 5 nm AuNPs that decrease 50% of A549 cells proliferation after 24 and 48 h measured by mLDH and MTT assay.

### 3.3 Cell Recovery upon Exposure to Different Functionalized AuNPs

**Figure 3:**
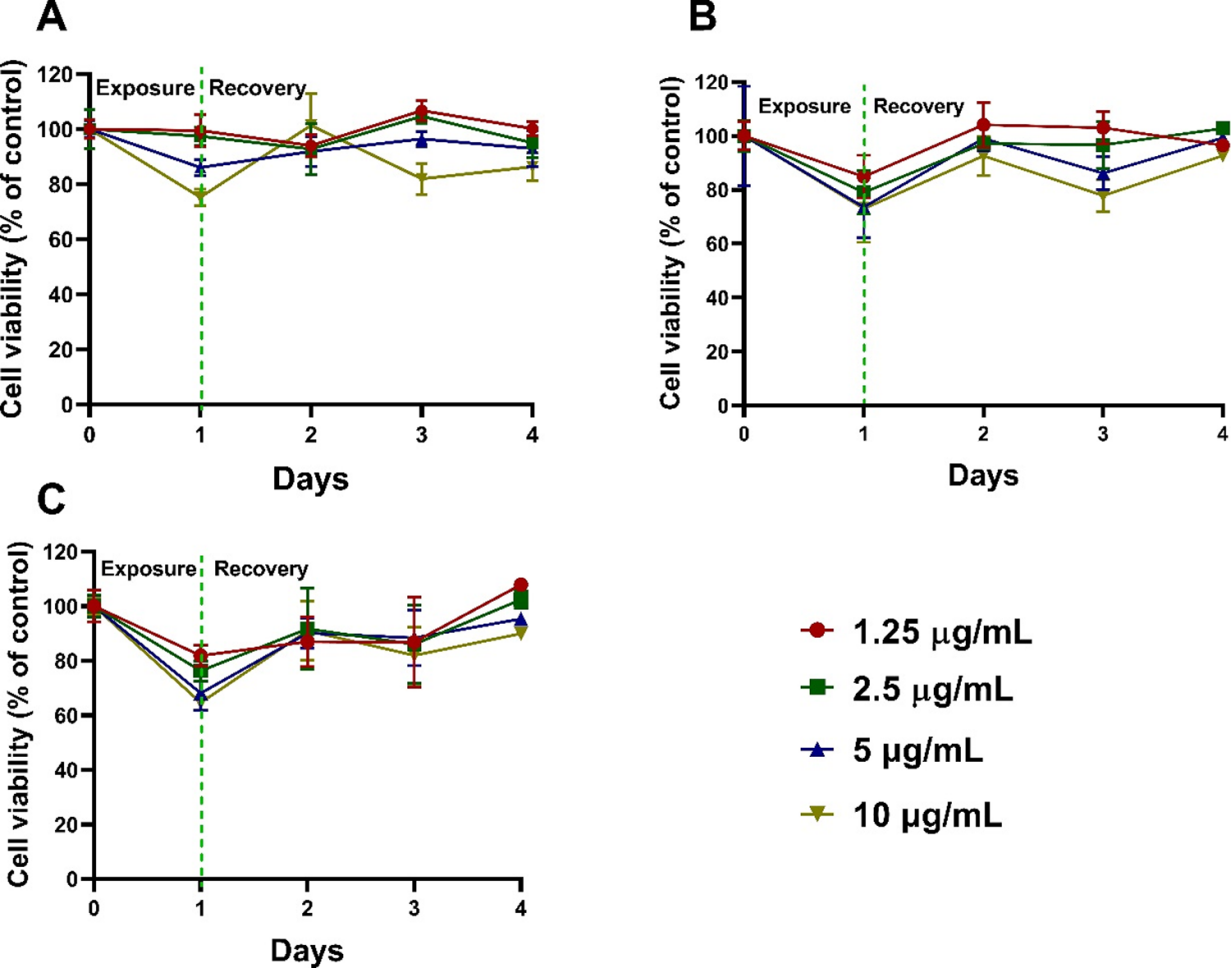
A549 cell recovery following exposure to functionalized 5 nm AuNPs. The cells were treated with AuNPs (1.25–10 μg/mL) for 1 day. Then, they were allowed to recover by changing their media on days 2, 3, and 4. The percentage cell viability was measured using mLDH. (A) Cell recovery after exposure to 5 nm tannate functionalized AuNPs. (B) Cell recovery following exposure to 5 nm citrate functionalized AuNPs. (C) Cell recovery after exposure to 5 nm PVP functionalized AuNPs. The plotted graphs represent the mean ± standard deviation of three independent experiments.

A549 cells to recover their proliferative state following AuNPs exposure was measured using the mLDH assay at a concentration range of 1.25–10 μg/mL which was selected based on the previous cell viability assay results. As shown in Figure 3A, the resumption of cell proliferation capacity was observed after the cells were treated with 1.25 μg/mL of 5 nm tannate functionalized AuNPs for 2 days. When incubated with 2.5 μg/mL, the cells recovered only at day four. In addition, treatment with 5 and 10 μg/mL did not allow for cell recovery from tannate functionalized AuNPs toxicity. In the case of 5 nm citrate functionalized AuNPs, the cells also recovered at days 2 and 3. Surprisingly, the cells were able to recover when incubated with 2.5 μg/mL at day 4 (Figure 3B). With the 5 nm PVP functionalized AuNPs, the cells were not able to recover to their proliferation state at any concentration on days 2 and 3. However, with 1.25 and 2.5 μg/mL, the cells slightly recovered at day 4 compared to untreated cells (Figure 3C).

### 3.4 Effects of Different Functionalized 5 nm AuNPs on Oxidative Stress

**Figure 4:**
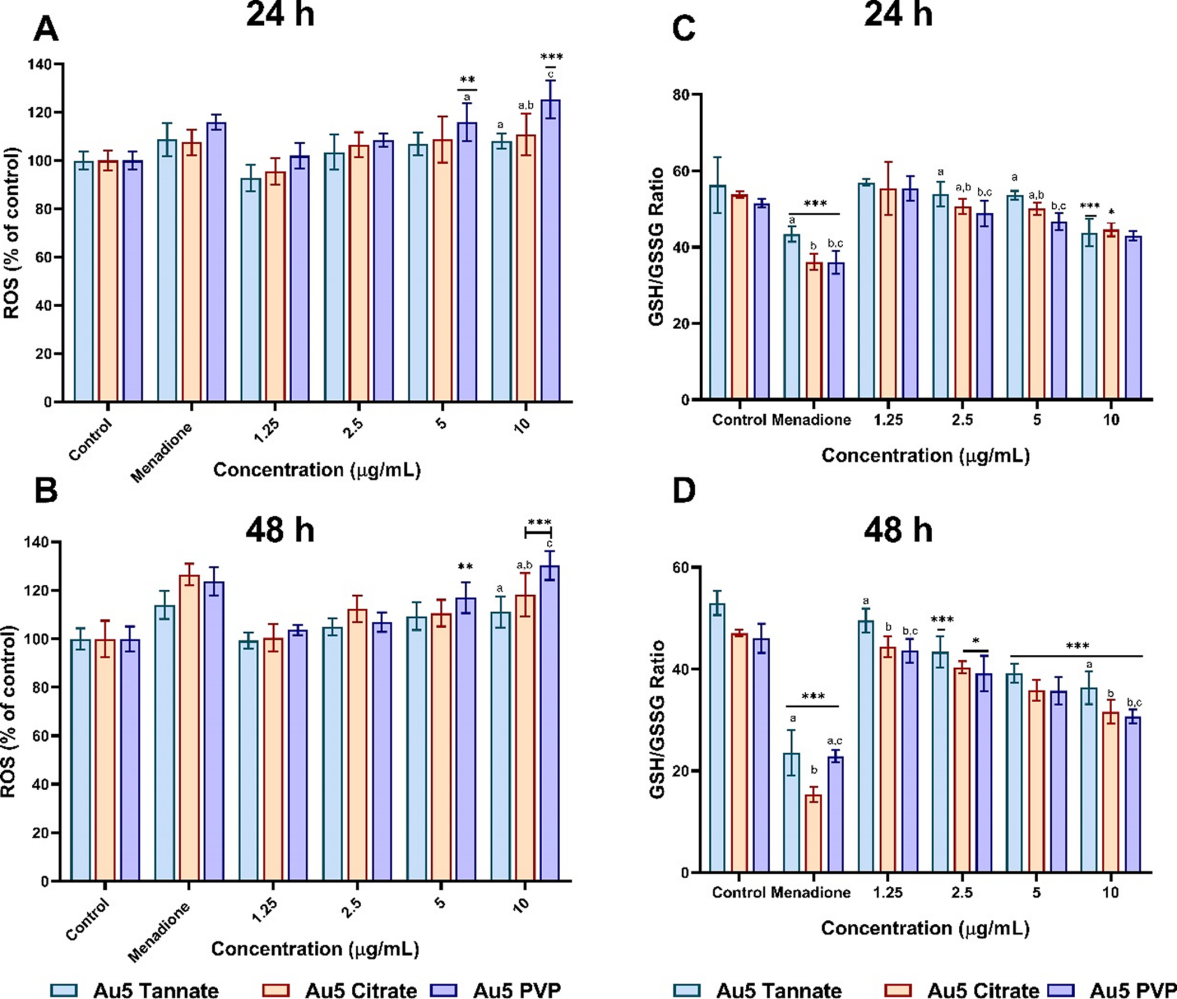
Oxidative stress induction in A549 cells after exposure to 5 nm functionalized AuNPs at a concentrations range of 1.25–10 μg/mL. Intracellular ROS induced after 24 h (A) and 48 h (B). GSH/GSSG ratio level after 24 h (C) and 48 h (D). Menadione (50 μM) was used as a positive control for 1 h. The plotted graphs represent the means ± standard deviation of five independent experiments. Bars with an asterisk (*) show statistical difference (*p < 0.05, **p < 0.01, and ***p < 0.001) when with control. Data with different letters above the bars represent the statistically significant difference (*p* < 0.05) between the functionalized AuNPs.

ROS generation can play a significant function in oxidative damage and apoptosis induction.^35−37^ To explore the effects of AuNPs in the induction of programmed cell death, the intracellular level of ROS was assessed in A549 cells after exposure to the three functionalized AuNPs for 24 and 48 h at a concentration range of 1.25–10 μg/mL with 50 μM of menadione used as a positive control for 1 h. As shown in Figure 4A,B, no difference was observed when A549 cells were treated at the lowest concentration (1.25 μg/mL) with all AuNPs after 24 and 48 h compared to the control. As the AuNPs concentration increased, the intracellular level of ROS evidently increased in a time-dependent manner in comparison to the control. Additionally, 5 nm PVP functionalized AuNPs showed a significant (*p* < 0.05) increase in ROS compared to their citrate and tannate counterparts.

Furthermore, the effects of functionalized 5 nm AuNPs were examined on intracellular glutathione levels in A549 cells. Glutathione plays a crucial role in maintaining cellular redox balance, cell proliferation, apoptosis, and signal transduction.^38,39^ A dose-dependent depletion of the GSH/GSSG ratio was observed after 24 h, as shown in Figure 4C. In addition, 10 μg/mL of all the AuNPs showed a reduction in the GSH/GSSG ratio to 80% when incubated for 24 h. Similarly, when cells were exposed to 2.5, 5, and 10 μg/mL of tannate functionalized AuNPs for 48 h, the GSH/GSSG ratio was reduced to 82, 75, and 69% respectively. A similar extent of reduction in the GSH/GSSG ratio was observed with citrate functionalized AuNPs after 48 h, as shown in Figure 4D. However, exposure of A549 cells to 5 nm PVP functionalized AuNPs for 48 h showed greater depletion of GSH/GSSG ratio to 78, 70, and 65% at a concentration of 2.5, 5, and 10 μg/mL respectively.

### 3.5 Effect of Functionalized AuNPs on MMP

**Figure 5:**
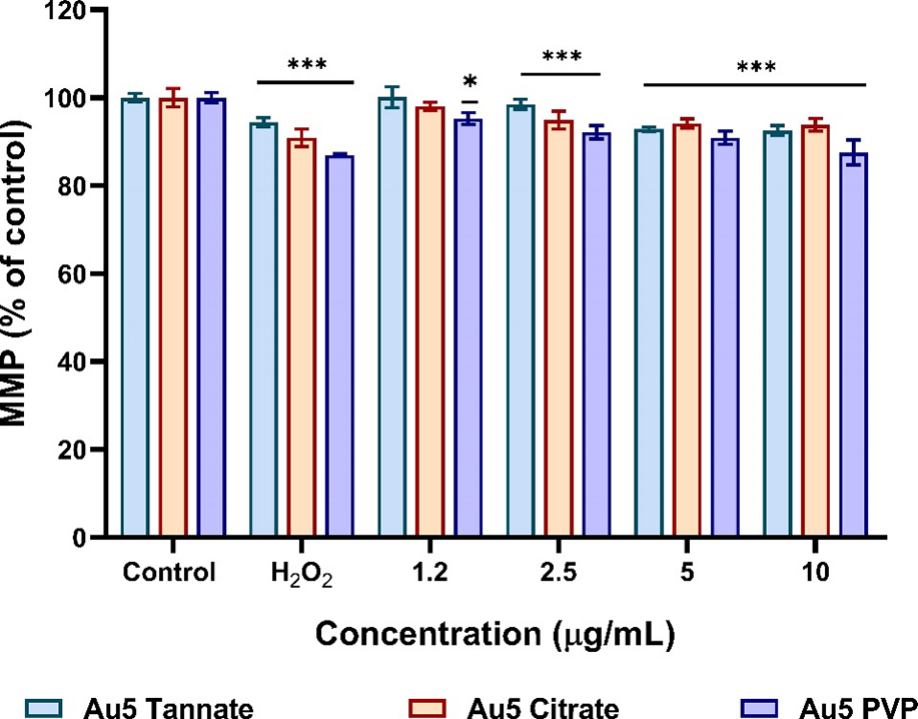
A549 MMP after exposure to 5 nm tannate, citrate, and PVP functionalized AuNPs at a concentration range of 1.25–10 μg/mL for 24 h assayed using an HCS mitochondrial health kit. Hydrogen peroxide (H_2_O_2_) (100 μM) was used as a positive control for 1 h. The plotted graphs represent the means ± SD of five independent experiments. Bars with an asterisk (*) show statistical differences (*p < 0.05, **p < 0.01, and ***p < 0.001) compared with the control.

To assess the effect of surface functionalized AuNPs on A549 MMP, depletion of MMP was measured using HCS Mitochondrial Health assay.^31^ As shown in Figure 5 a slight decrease in MMP was observed in A549 cells after 24 h incubation with all the AuNPs concentration range of 1.25–10 μg/mL. At the highest concentration (10 μg/mL), PVP functionalized AuNPs caused a slight decrease (87%) in MMP, followed by tannate (92%) and citrate functionalized AuNPs (93%). On the other hand, incubation of A549 cells with the positive control (H_2_O_2_) showed a much greater decrease in MMP compared to the control and AuNP treatments. Similar observations were confirmed with fluorescence intensity images at the highest concentration of PVP functionalized AuNPs compared to untreated cells (Figure S3 in the Supporting Information).

### 3.6 Apoptosis Induction by Different Functionalized AuNPs

**Figure 6:**
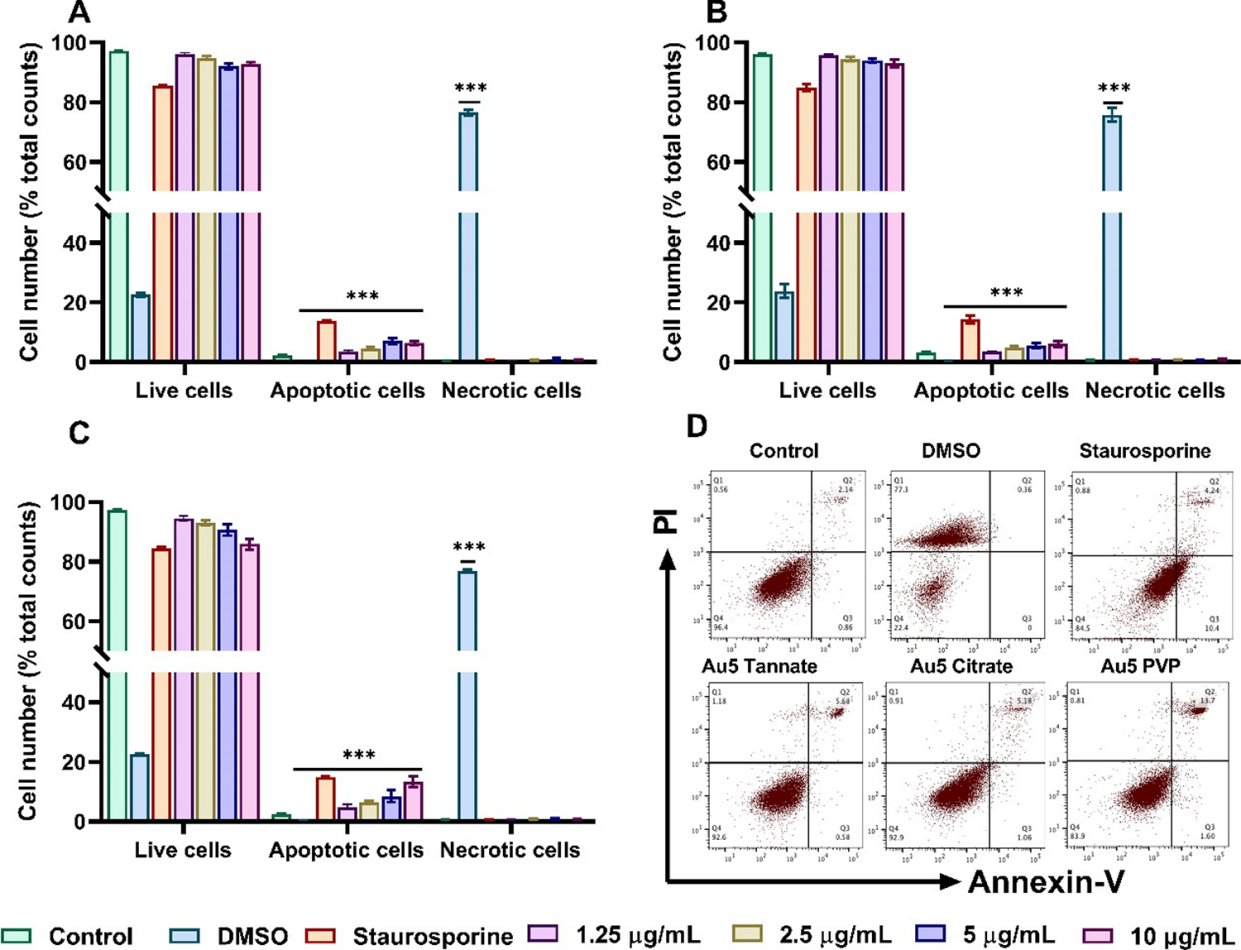
Flow cytometric analysis of A549 cells exposed to 1.25–10 μg/mL of different functionalized 5 nm AuNPs for 24 h stained with Annexin V and PI. (A) A549 cells treated with tannate functionalized AuNPs. (B) A549 cells treated with citrate functionalized AuNPs (C) A549 cells treated with PVP functionalized AuNPs. (D) Dot plots analysis of apotosis incubated with 10 μg/mL of tannate, citrate, and PVP functionalized AuNPs. Treatment with 1 μM staurosporine and 10% DMSO for 6 h was used as a positive control for apoptosis and necrosis. The plotted graphs represent the means ± standard deviation of three independent experiments. Bars with an asterisk (*) show a statistical difference (***p < 0.001) compared with the control.

Given that ROS generated by AuNPs can cause damages to membrane organelles which can consequently lead to the activation of apoptosis in A549 cells.^40,41^ The correlation between the ROS generation and apoptosis induction by AuNPs was investigated using Annexin V and PI staining assay. As shown in Figure 6A, 10 μg/mL tannate functionalized AuNPs increased the percentage of apoptosis to 6.37 ± 0.61% which was significantly greater (*p* < 0.001) than control (2.34 ± 0.20%). Similarly, citrate functionalized AuNPs were found to induce apoptosis by 6.13 ± 0.97% with the highest concentration of 10 μg/mL (Figure 6B). Interestingly, at the same concentration, 5 nm PVP AuNPs showed the highest percentage of apoptotic cells (13.39 ± 1.71%) (Figure 6C). In addition, FlowJo scatter dot plots clearly highlighted that the percentage of apoptotic cells in A549 cells incubated with 10 μg/mL of PVP AuNPs is the highest compared to their tannate and citrate functionalized counterparts (Figure 6D). This suggests that PVP-AuNPs could suppress A549 cell proliferation through the induction of apoptosis.

### 3.7 Determining the Expression Levels of DUBs, PI3K/AKT/mTOR, and Wnt Signaling Related Proteins in Noncancerous and Cancerous Lung Cell Lines without AuNPs

**Figure 7:**
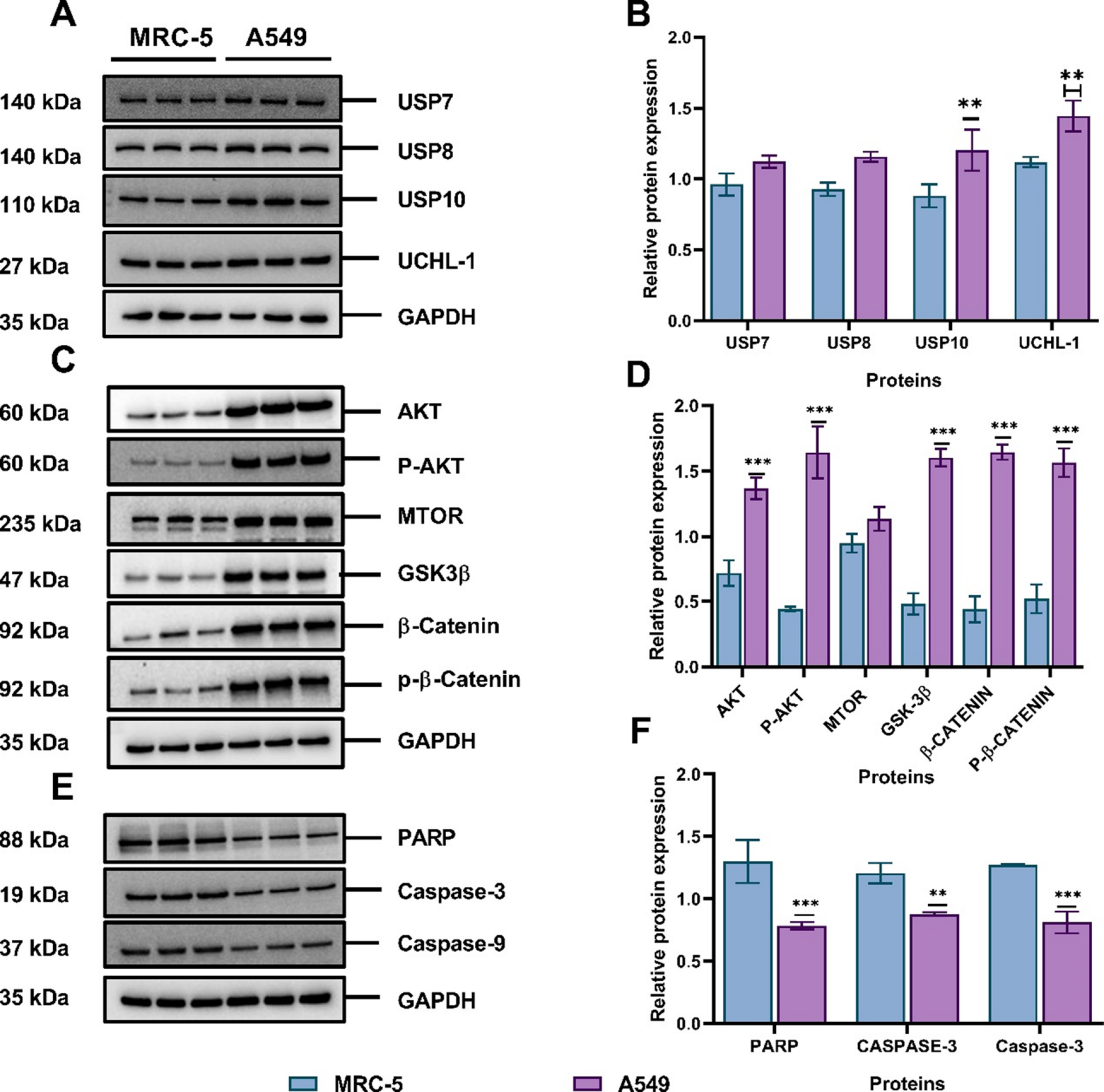
Baseline protein expression in noncancerous MRC-5 and cancerous A549 cells detected by Western blot and semiquantified using ImageJ. (A) and (B) DUBs protein expression level; (C) and (D) PI3K/AKT/mTOR and Wnt signaling pathway related proteins; (E) and (F) apoptosis-related proteins expression. The plotted graphs represent the mean ± SD (*n* = 3). Bars with an asterisk (*) show statistical difference (**p < 0.01 and ***p < 0.001) when A549 cells compared to MRC-5.

In order to explore the potential effects of the functionalized AuNPs on different intracellular protein levels, the expression of DUBs, PI3K/AKT/mTOR, and Wnt related proteins were initially assessed in A549 cells and noncancerous lung fibroblast (MCR5) to determine their baselines without AuNPs. Western blotting was used to determine those levels, and images were semiquantified using ImageJ software. Interestingly, all the investigated proteins were highly upregulated in the cancerous A549 cells compared to noncancerous MRC-5 cells, as shown in Figure 7A–C. In order to easily compare the Western blot bands, the levels of proteins were semiquantified using ImageJ. The latter highlighted that some proteins (USP7, USP8, USP10, UCHL-1, AKT, p-AKT, mTOR, β-catenin, and p-β-catenin) were upregulated in A549 cells after 24 h, which was statistically significant compared to MRC-5 cells (Figure 7B–D). However, the expression levels of apoptotic proteins (PARP, caspase-3, and caspase-9) were downregulated in A549 cells compared to noncancerous lung fibroblast (MRC-5), as shown in (Figure 7E,F).

### 3.8 Effects of Different Functionalized AuNPs on DUBs Expression in A549 Cell Lines

**Figure 8:**
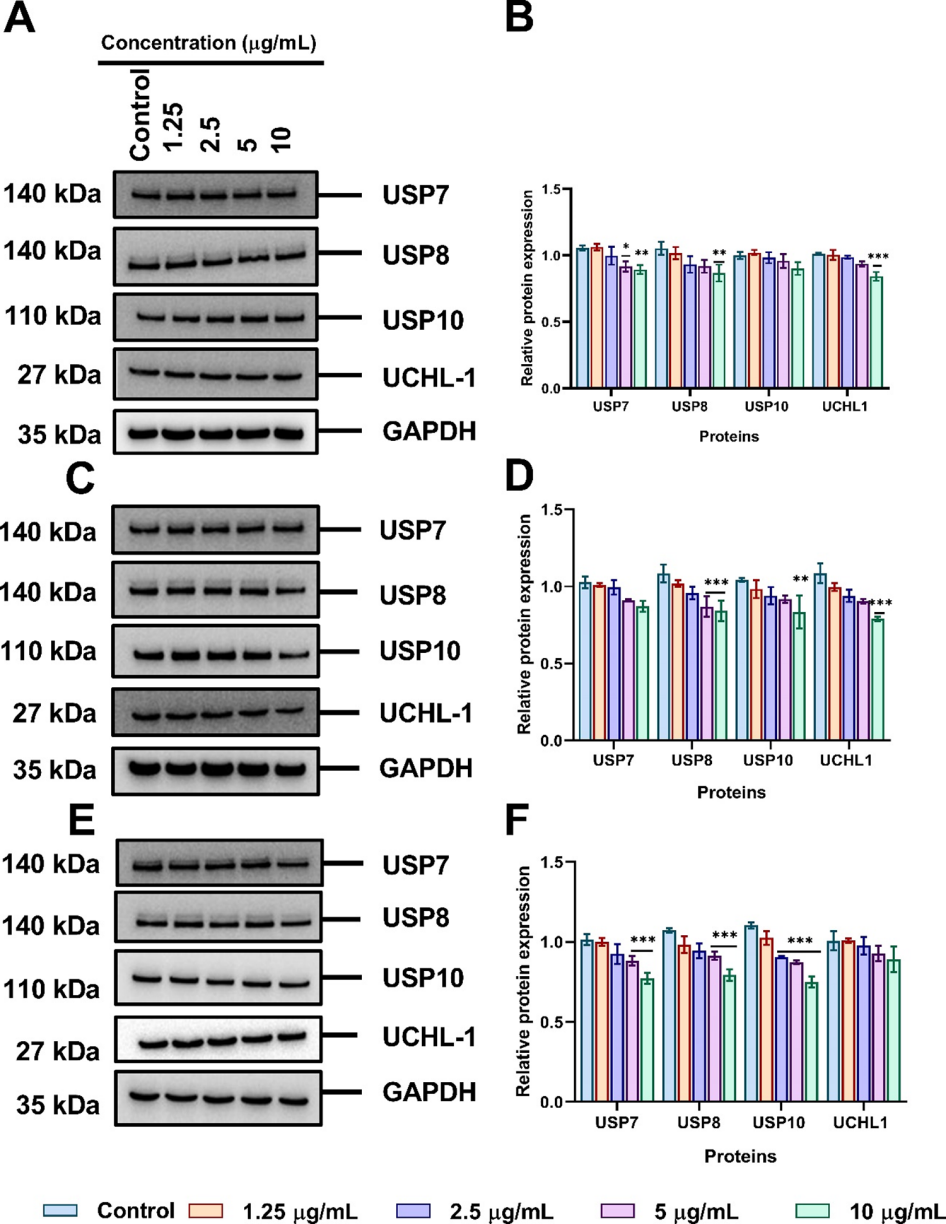
Surface functionalized AuNPs inhibit deubiquitinating enzymes (DUBs) in A549 cells treated with a concentration of 1.25–10 μg/mL for 24 h detected by Western blotting and semiquantified using ImageJ. DUBs expression exposed to tannate functionalized AuNPs (A) and (B), citrate functionalized AuNPs (C) and (D), PVP functionalized AuNPs (E) and (F). The plotted graphs represent the means ± SD of three independent experiments. Bars with an asterisk (*) show statistical differences (*p < 0.05, **p < 0.01, and ***p < 0.001) compared with the control.

To investigate the effects of functionalized AuNPs on the ubiquitin proteasome system (UPS), protein levels of deubiquitinating enzymes such as USP7, USP8, USP10, and UCHL-1 (involved in ubiquitin removal during the ubiquitination process) were investigated following treatment with the different surface functionalized AuNPs for 24 h using Western blotting. As shown in Figure 8A, only 10 μg/mL of tannate functionalized AuNPs affected protein levels of USP7, USP8, and USP10 when incubated for 24 h. However, the level of UCHL-1 remained unchanged after 24 h but was followed by a slight decrease after incubation 10 μg/mL. The semiquantification of protein levels using ImageJ demonstrated that the effects were more pronounced at 10 μg/mL, as shown in Figure 8B. Similarly, citrate functionalized AuNPs slightly reduced the expression of USP7, USP8, and USP10 at 10 μg/mL but there was a significant change in the total protein level of UCHL-1 in comparison to the control (*p* < 0.001) (Figure 8C,D). Likewise, AuNPs functionalized with PVP slightly downregulated the levels of USP7, USP8, USP10, and UCLH-1 in A549 cells with 10 μg/mL (Figure 8E,F).

### 3.9 Effects of Functionalized AuNPs on Proteins in the PI3K/AKT/mTOR and Wnt Signaling Pathways in A549 Cell Lines

**Figure 9:**
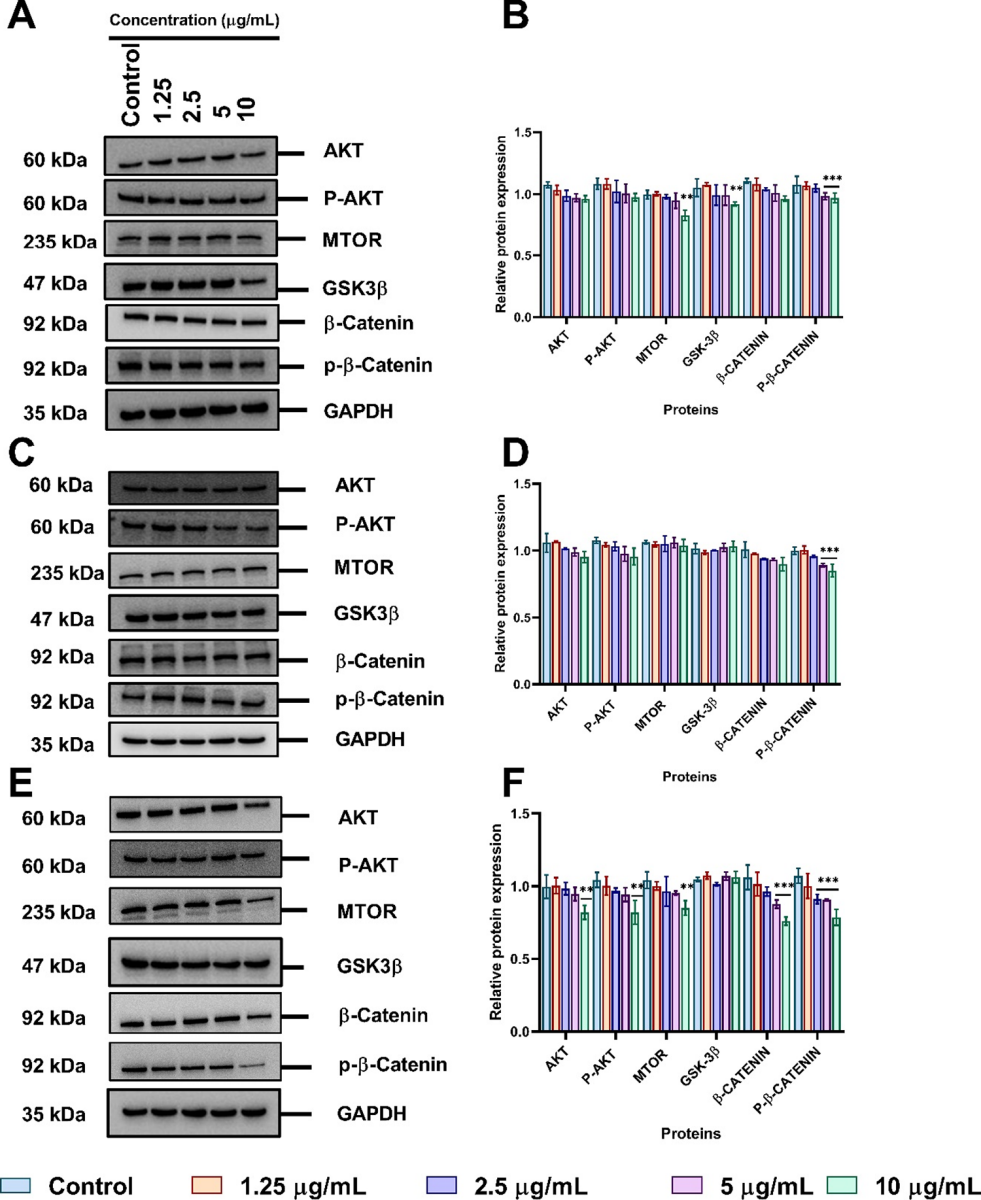
Surface functionalized AuNPs inhibit PI3K/AKT/mTOR and Wnt related proteins in A549 cells treated with a concentration range of 1.25–10 μg/mL for 24 h analyzed by Western blotting and semiquantified using ImageJ. PI3K/AKT/mTOR and Wnt signaling proteins expression exposed to tannate functionalized AuNPs (A, B), citrate functionalized AuNPs (C, D), (E, F) PVP functionalized AuNPs. The plotted graphs represent the means ± SD of three independent experiments. Bars with an asterisk (*) show statistical difference (*p < 0.05, **p < 0.01, and ***p < 0.001) when compared with the control.

To further explore the effects of AuNPs on intracellular signaling pathways following the inhibition of DUBs, the expression of AKT, p-AKT, mTOR, GSK-3β, β-catenin, and p-β-catenin proteins was evaluated upon exposure to different functionalized AuNPs. As shown in Figure 9A, tannate functionalized AuNPs significantly affected the level of AKT, p-AKT, mTOR, β-catenin, and p-β-catenin in a dose- and time-dependent manner. However, GSK-3β protein levels slightly increased at 24 h but decreased as the concentration increased to 10 μg/mL compared to control. Relative protein expression as semiquantified by ImageJ (Figure 9B) confirmed that protein levels of AKT, p-AKT, mTOR, β-catenin, and p-β-catenin in A549 cells were indeed significantly (*p* < 0.001) downregulated with 10 μg/mL. Interestingly, similar downregulation of proteins was observed with both citrate (Figure 9C,D) and PVP functionalized AuNPs in A549 cells (Figure 9E,F).

### 3.10 Effect of Functionalized AuNPs on the Expression of PARP, Caspase-3, and Caspase-9 in A549 Cell Lines

**Figure 10:**
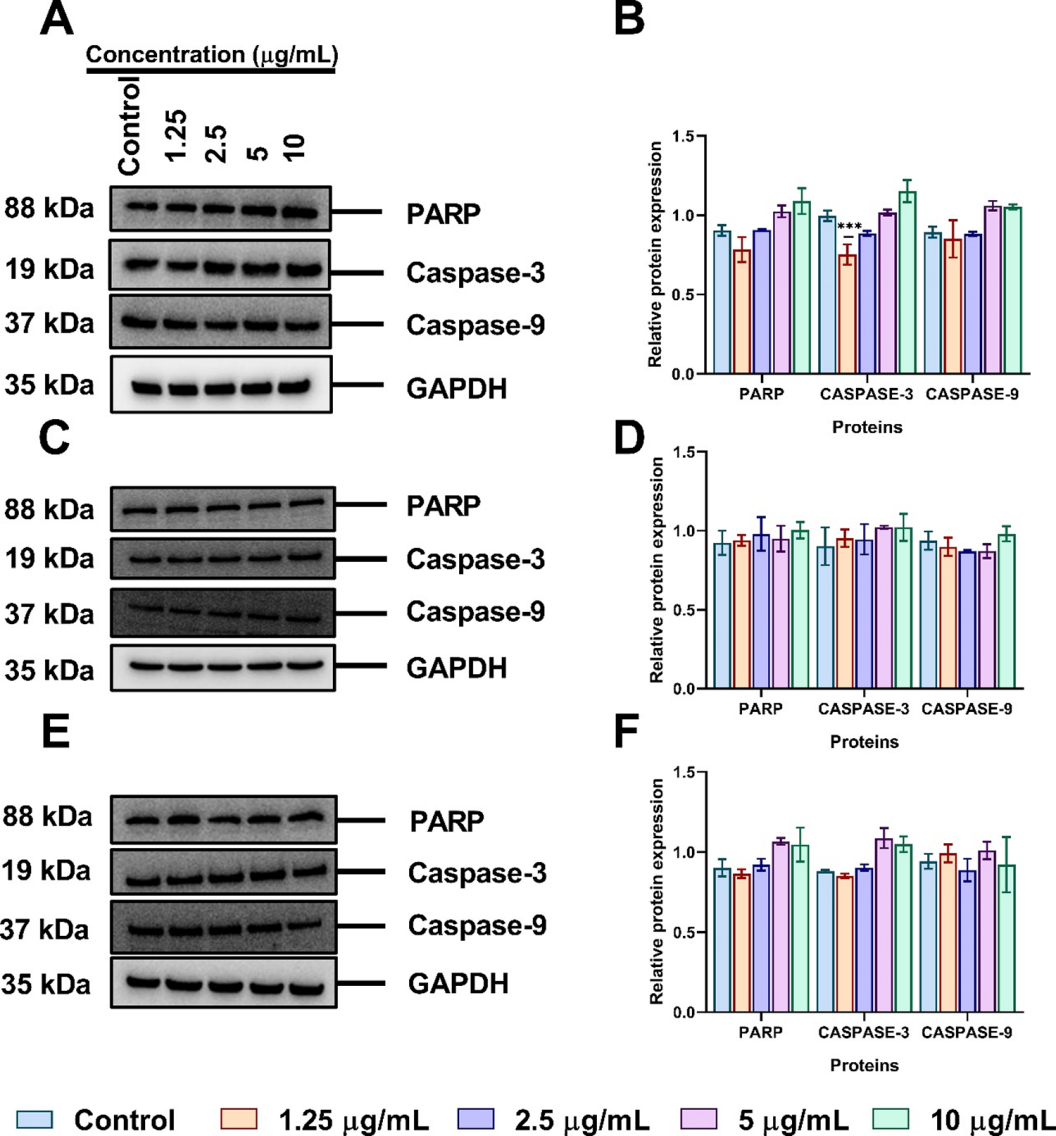
Surface functionalized AuNPs induced mitochondria-related apoptosis in A549 cells treated with concentrations of 1.25–10 μg/mL for 24 h assessed by Western blotting and semiquantified using ImageJ. PARP, caspase-3, and caspase-9 protein expression after treatment with tannate functionalized AuNPs (A, B), citrate functionalized AuNPs (C, D), and PVP functionalized AuNPs (E, F). The plotted graphs represent the means ± SD of three independent experiments. Bars with an asterisk (*) show statistical difference (*p < 0.05, **p < 0.01, and ***p < 0.001) when compared with the control.

To determine whether the different functionalized AuNPs also affected some proteins which are crucial in apoptosis; the expression of PARP, caspase-3, and caspase-9 was assessed using Western blotting. As shown in Figure 10A,B, treatment with tannate functionalized AuNPs upregulated the expression of PARP, caspase-3, and caspase-9 in a dose-dependent manner after 24 h. Likewise, the expression of PARP and Caspase-3 protein levels were also observed to be upregulated with citrate functionalized AuNPs after 24 h (Figure 10D,C). Interestingly, PVP functionalized AuNPs only affected caspase-9 protein expression at 10 μg/mL when compared to the untreated control while the expression levels of PARP and caspase-3 showed a dose- and time-dependent increase after treatment (Figure 10E,F).

## 4 Discussion

Research on AuNPs has received enormous attention in the field of nanotechnology due to their intrinsic properties which have shown great usage in biomedical applications such as tumor imaging and drug delivery.^42,43^ A549 lung cells have been used as models for the study of the drug delivery potential of several NPs.^44−46^ Thus, they have been used in this work as an in vitro model to investigate the anticancer potential of different functionalized AuNPs. Tannate, citrate, and PVP were used as functionalized ligands in this study due to the high tendency of AuNPs to aggregate in solution. When modified or stabilized using these organic ligands, they cause steric repulsion and prevent aggregation of AuNPs due to their hydrophilic chain structure.^47,48^ The variability of the functionalizing agents or ligands attached to the AuNP surface determines their cellular uptake by the cells as well as cytotoxicity.^49,50^ Prior to cytotoxicity assessment, various characterization techniques were used to assess the size, PDI, and zeta potential of AuNPs in different biological milieu and morphology in UPW. The size distribution obtained when the AuNPs were dispersed in CCM was more than that in PBS, FCM, and UPW. This size discrepancy occurred because AuNPs are easily aggregated in CCM due to the high ionic nature of the solution and the proteins which may cause the particles to increase in size.^51^ The high concentrations of ions in the CCM can decrease the screen length of the charged chemical group on the AuNPs, leading to the thermodynamically preferred replacement of surface-related molecules with serum proteins.^12,52^ Unwanted aggregation of AuNPs can presumably affect their degree of uptake and biological activity in the cells.^53^ Zeta potential measurements revealed that all the functionalized AuNPs have negatively charged surfaces with zeta values greater than ±5 mV. This indicates that the particles have enough electrostatic repulsion to remain stable in an aqueous solution. In addition, the charge on AuNPs could be affected by the surrounding environment. For instance, the zeta potential of AuNPs in CCM became less negative than that in water (around −7 mV) independent of functionalization due to charge neutralization and interaction with the serum proteins in the media. This suggests that the dispersion of AuNPs into the CCM or deposition in the cells would lead to the formation of protein corona and a slight change in the surface of functionalized AuNPs.^31,54,55^ Moreover, morphological observation using TEM revealed that AuNPs functionalized with tannate were spherical in shape with a slight aggregation, while the AuNPs functionalized with citrate and PVP images were monodispersed with an excellent dispersion. This suggests that the functional group of the surface functionalization agent could be the driver for AuNP aggregation.^56,57^ mLDH and MTT assays were then used to determine percentage cell viability as an indication of the cellular response of A549 cells to AuNPs.^29^ The mLDH and MTT assays highlighted that citrate, PVP, and tannate functionalized AuNPs significantly decreased cell viability and metabolic activity respectively in a dose- and time-dependent manner. Furthermore, PVP functionalized AuNPs showed a significant (*p* < 0.05) decrease in cytotoxicity in A549 cells compared to citrate and tannate functionalized AuNPs as well as excellent IC_50_ values. The differences in toxicity and IC_50_ values could be attributed to the functionalization agents and zeta potentials charge. PVP functionalized AuNPs show a less negatively charged surface compared to others and may yield stronger electrostatic interactions between PVP–AuNP and the negatively charged biological membrane surface. This could ultimately determine their cellular uptake fate as well as their rate of cytotoxicity.^58,59^ Similar to the findings presented here, several studies have highlighted the influence of functionalization on the toxicity of AuNPs.^60−62^ They found that AuNPs functionalized with a positive charge have a stronger binding affinity for the cell membrane, thereby promoting their uptake and toxicity compared to AuNPs functionalized with a negative or neutral charge. Although negatively charged functionalized AuNPs were used in this study, it was still observed that the toxicity of AuNPs does not only depend on the surface charge of the functionalized AuNPs but also on the functionalizing agents used during the synthesis. In addition, when cells were exposed to a high concentration of functionalized AuNPs for 24 and 48 h, the proliferation of A549 cells relatively decreased. The decrease in cell proliferation at higher concentrations of AuNPs could be mediated by the insufficient nutrient provided in the CCM and more available number of AuNPs to interact with cellular components such as proteins, fatty acids, nucleic acid, and carbohydrates.^23,63,64^ Based on the cell viability results, the capacity of A549 cells to recover from the detrimental effects caused by AuNPs was studied. Results on cell recovery demonstrated that A549 cells have the capacity to return to normal proliferation at low AuNP concentrations. However, the cell viability continued to decline at higher concentrations. The interference of the higher concentrations with cell proliferation is probably due to both AuNPs and the ions released by dissolution. Previous studies have also reported that cells can return to their normal state if not compromised by mechanical damage such as massive internalization.^65−67^ Given that ROS is directly related to AuNPs cytotoxicity, the level of ROS was measured after 24 and 48 h incubation with 1.25–10 μg/mL of the three different functionalized AuNPs in A549 cells. ROS can dynamically influence the tumor microenvironment survival, proliferation, and metastasis by enhancing intracellular oxidative stress.^68,69^ Consequently, a potent method of combating cancer may be realized by disrupting the equilibrium of intracellular ROS to cause their elevation.^70^ In this study, an elevated level of intracellular ROS was observed when the cells were treated with high concentrations of different functionalized AuNPs compared to untreated cells. The possible generation of ROS in A549 cells could be either by the direct AuNP induction of ROS via a Fenton-like reaction or an indirect reaction between the released metal ions and a thiol moiety on the respiratory chain enzyme inside the mitochondria.^71,72^ Activation of nicotinamide adenine dinucleotide phosphate oxidase that converts NADPH into NADP^+^, as well as cytochrome c oxidation in the mitochondria, can both contribute to an increase in ROS elevation by functionalized AuNPs.^73^ Likewise, intracellular glutathione levels were significantly decreased in A549 cells in a dose- and time-dependent manner after exposure to the three functionalized AuNPs. The elevation of intracellular ROS along with the depletion of intracellular glutathione suggests that oxidative stress induction may be the primary mechanism for the toxicity of functionalized AuNPs in A549 cells. This agrees with previous studies, which demonstrated that functionalized AuNPs might induce cytotoxicity through ROS production and the depletion of intracellular glutathione.^42,74^ Since excessive production of ROS in the cells is associated with mitochondrial damage,^75^ the MMP induced by different functionalized AuNPs was further investigated. Evidence from the results revealed that functionalized AuNPs altered mitochondrial functions through a decrease in MMP which was more pronounced with PVP functionalized AuNPs. This observation indicates that the surface functionalization of AuNPs has a crucial influence on their mitochondrial effect. Previous results showed that mitochondrial stress and a decrease in MMP occur in different cell types following treatment with functionalized AuNPs.^76,77^ Loss of MMP coupled with the increase in the production of ROS in the cells may trigger the initiation of mitochondrial apoptosis by cytochrome c released into the cytoplasm which could result in the activation of the caspase cascade.^78,79^ The results of this study revealed that all the functionalized AuNPs increased the number of apoptotic cells in a concentration-dependent manner with PVP-AuNPs showing slightly higher induction. This correlates with cell viability or cell membrane damage, MMP, and ROS analysis that show that at the same concentration, PVP functionalized AuNPs have greater effects on A549 cells compared with AuNPs functionalized with citrate and tannate. Several studies have suggested that the most predominant mechanism of cytotoxicity through which AuNPs suppress the proliferation of cancer cells is apoptosis.^80,81^ For instance, the percentage of apoptotic cells was demonstrated to increase when A549 cells were treated with citrate functionalized 5 nm AuNPs compared to 5 nm polyethylene imine functionalized AuNP, confirming that there could be functionalization-dependent cell death.^15^ Elsewhere, the number of apoptotic cells was reported to gradually increase when HepG2 liver cells were treated with polyethylene glycol (PEG) and lithocholic acid functionalized AuNPs.^14^ Their observations revealed that when the functionalized AuNPs are absorbed by the cells, they release Au ions which may lead to mitochondrial dysfunction and oxidative stress through ROS accumulations, ultimately leading to apoptosis.

Next, the effect of surface functionalization on deubiquitinating enzymes (DUBs) expression in A549 cells was evaluated using Western blotting. This was performed to investigate the anticancer mechanism of functionalized AuNPs and correlate the critical end points finding (cytotoxicity, ROS, MMP) with that of DUB protein expression. Several studies have shown that targeting DUBs might be an effective approach in cancer therapy due to their significant role in controlling cellular mechanisms such as apoptosis, cell proliferation, DNA transcription and repair, cell cycle progression, immune response, and protein modification.^82−85^ In this study, the protein expression of DUBs in cancerous A549 and noncancerous MRC-5 lung fibroblast cells without AuNP treatment was first analyzed. It was found that proteins were upregulated in A549 cells compared to MRC-5 which correlated with the proliferation of A549 cells being cancerous cells. Interestingly, when A549 cells were treated with 1.25–10 μg/mL of different surface functionalized AuNPs, the protein expression levels of DUBs in A549 cells significantly decreased with increasing concentration. The downregulation in the expression of DUBs could be related to surface functionalization agents which are responsible for AuNP interactions with the biological membrane as well as their interaction with specific proteins. This is the first attempt to study the effects of functionalized AuNPs on the ubiquitin proteasome system enzymes of A549 cells, and other studies are urgently needed to confirm the evidence. However, our previous study showed that AuNPs (5, 10, and 80 nm) were able to induce size-dependent cytotoxicity to A549 cells by inhibiting DUBs such as USP7, USP8, USP10, and UCHL-1.^23^ Other studies have also reported that small molecules like P5091, HBX19818, P22077, b-AP15,, and WP1130 can attenuate the proliferation of different cell lines through the inhibition of ubiquitin-specific proteases such as USP7,^86−89^ USP8,^90−92^ USP10,^93−96^ USP14,^97,98^ and Ubiquitin C-terminal hydrolases such as UCHL5.^98^ We further investigated if the downregulation of DUBs can modulate the expression of AKT and Wnt signaling proteins after exposure to different functionalized AuNPs. A decrease in the expression of AKT and Wnt signaling-related proteins was observed when compared with untreated cells. Studies have shown that functionalized AuNPs inhibit the proliferation of cancer cells through the modulation of the PI3K/AKT/mTOR pathway.^10,35^

The most common mechanism through which NPs inhibit the proliferation of cancer cells is through the induction of apoptosis.^10,99−103^ This current study then explored if the exposure of three functionalized AuNPs induced apoptosis by analyzing the mitochondrial expression of PARP, caspase-3, and caspase-9 using Western blotting. During apoptosis, these proteins are activated and play a significant role in the execution of mitochondrial-dependent apoptosis.^100,104^ The findings demonstrated that all the functionalized AuNPs induced mitochondrial-related apoptosis in a dose-dependent manner by increasing the expression of PARP, caspase-3, and caspase-9 proteins, which corresponds to apoptosis induction by AuNPs.^46,80^

## 5 Conclusions

This study showed that all three functionalized AuNPs induced reduction in cell viability of A549 cells through decrease in mitochondrial activity, cell membrane integrity, glutathione, mitochondrial membrane potential depletion, and an increase in ROS production. In-depth analysis demonstrated that 5 nm functionalized AuNPs might affect the USP by partially inhibiting the expression level of DUBs. Furthermore, the results demonstrated that AuNPs may induce mitochondrial-related apoptosis in A549 cells through PI3K/AKT/mTOR and Wnt signaling pathways by decreasing the expression of related proteins which could be associated with the production of ROS. This study showed the importance of understanding the mechanism through which functionalized AuNPs are interacting with DUBs enzymatic pathways, thereby altering the cell functioning. This alteration in cell function and their mechanistic understanding has given a new insight into the anticancer properties of functionalized AuNPs. The study warrants a need for future work to investigate the effect of functionalized AuNPs on DUBs on other cancer cell types both in vitro and in vivo.

## Supporting Information Available

Absorption spectra of 5 nm functionalized AuNPs dispersed in UPW measured by UV–vis; cell viability of A549 cells exposed to stabilizing agents at 0.125–1 mM for 24 h; and fluorescence intensity images of A549 cells treated with different functionalized AuNPs at a concentration of 1.25–10 μg/mL stained with the mitohealth staining kit (PDF)
