# Supplementary material for: Functionalized Gold Nanoparticles Suppress the Proliferation of Human Lung Alveolar Adenocarcinoma Cells by Deubiquitinating Enzymes Inhibition
Source: ACS Omega. 2023 Oct 20;8(43):40622–38. doi: 10.1021/acsomega.3c05452 (PMC10620884; doi:10.1021/acsomega.3c05452)
Supplement: Supplementary file 1 — ao3c05452_si_001.pdf [file ao3c05452_si_001.pdf]

Supplementary Information

Functionalised Gold Nanoparticles Suppress the Proliferation of Human Lung Alveolar Adenocarcinoma Cells by Deubiquitinating Enzymes Inhibition.

Bashiru Ibrahim[a,b], Taiwo Hassan Akere[a,b], Swaroop Chakraborty[b]*, Eugenia Valsami-Jones[b]* and Hanene Ali-Boucetta[a]*

[a]Nanomedicine, Drug Delivery & Nanotoxicology (NDDN) Lab, School of Pharmacy, College of Medical and Dental Sciences, University of Birmingham, Birmingham B15 2TT United Kingdom
[b]School of Geography, Earth and Environmental Sciences, College of Life and Environmental Sciences, University of Birmingham, Birmingham B15 2TT United Kingdom

*h.aliboucetta@bham.ac.uk ; e.valsamijones@bham.ac.uk; s.chakraborty@bham.ac.uk

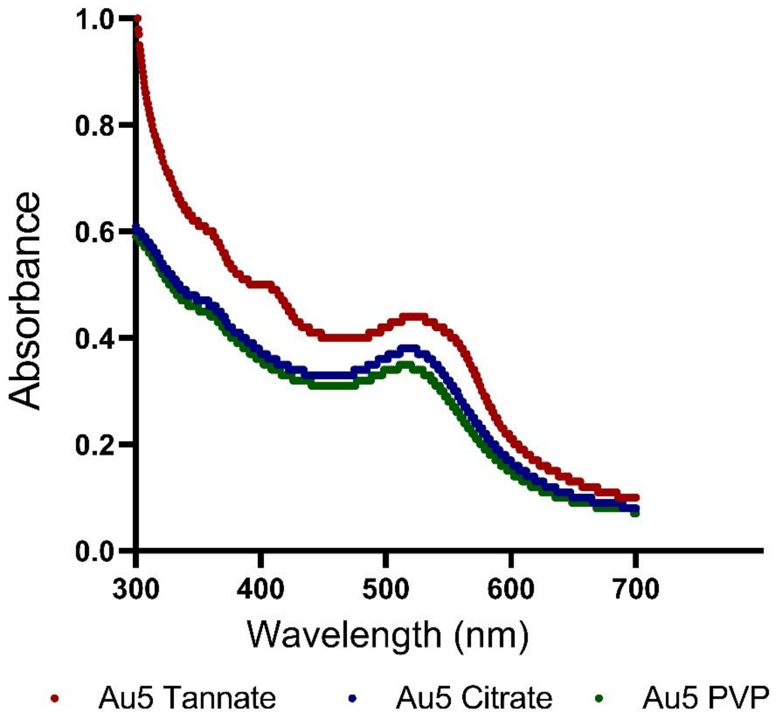

***Figure S1**: Absorption spectra of 5 nm AuNPs dispersed in UPW measured by UV-Vis.*

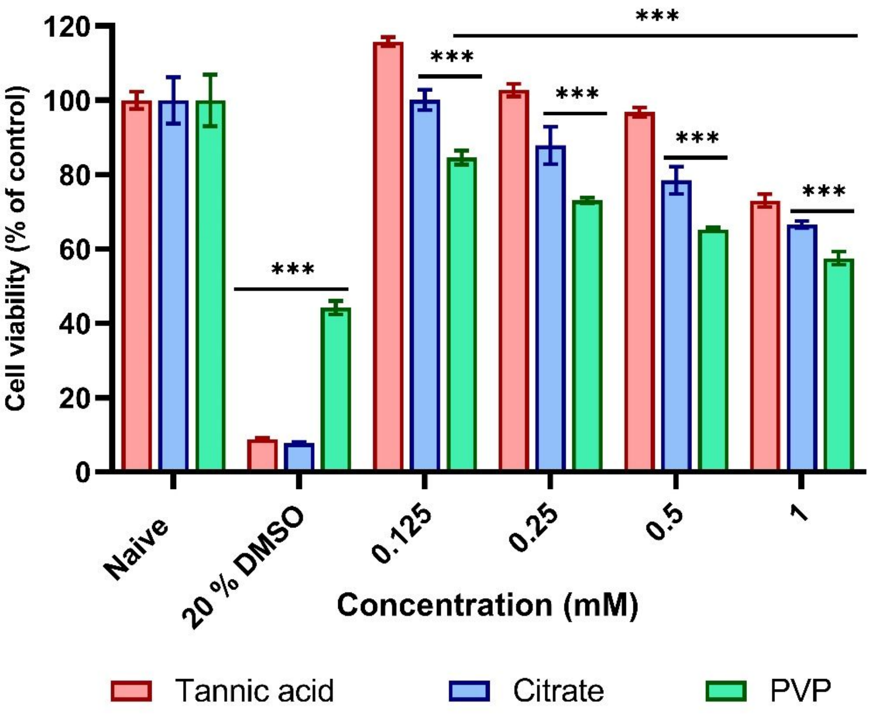

Figure S2: *Cell viability of A549 cells exposed to stabilising agents at 0.125-1 mM for 24 h. Data represent mean + standard deviation of 3 independent experiments. Bars with asterisk (*) show statistically different of (***P<0.001) when comapred with the control.*

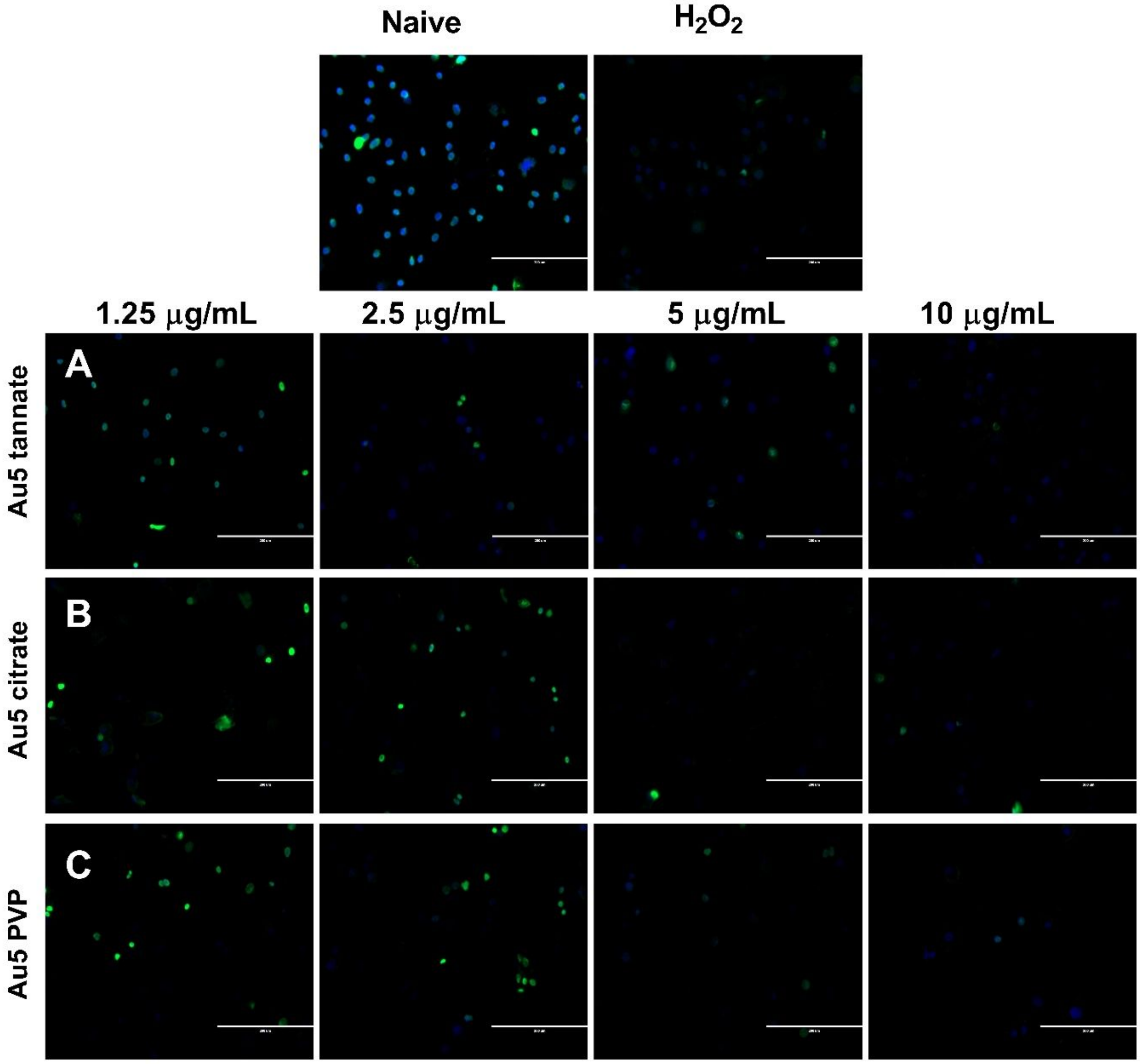

Figure S3: *Mitochondrial membrane potential of A549 cells treated with different functionalised AuNPs at concentration of 1.25 -10 µg/mL stained with mitohealth staining kit. Blue stainin indicate cell nuclues and the grean fluorensce shows mitohealth dye in the mitochondria. Fluorescence intensity of 5 nm AuNPs capped (**A**) Tannate (**B**) Citrate (**C**) PVP. The images were captured with an EVOS fluorescence microscope using 20X maganification. Scale bar (200 µM).*
